# Hearing Care Access Amidst Dementia in Primary Care Practice: Identifying Opportunities to Improve Integration

**DOI:** 10.1093/geroni/igaf122.3783

**Published:** 2025-12-31

**Authors:** Danielle Powell, Ariella Sapoznick, Afia Obeng, Christina Koch, Stephanie Nothelle, Esther Oh, Nicholas Reed, Jennifer Wolff

**Affiliations:** University of Maryland, University of Maryland College Park, Maryland, United States; University of Maryland, University of Maryland College Park, Maryland, United States; University of Maryland, University of Maryland College Park, Maryland, United States; University of Maryland Medical System, University of Maryland Medical System, Maryland, United States; Johns Hopkins School of Medicine, Johns Hopkins School of Medicine, Maryland, United States; Johns Hopkins School of Medicine, Johns Hopkins School of Medicine, Maryland, United States; New York University Grossman School of Medicine, New York, New York, United States; Johns Hopkins University - School of Public Health, Baltimore, Maryland, United States

## Abstract

Older adults with hearing loss and dementia face unique communication and care challenges which may inhibit navigation of the hearing care process. Current care models rarely equip primary care providers to initiate hearing care, especially for patients managing dementia. Few interventions have been co-designed with input from persons with dementia, care partners, and providers to understand how hearing needs are addressed outside the audiology clinic. We conducted thematic analysis of semi-structured interviews with primary care providers (n = 6), older adults with hearing loss and self-reported dementia (n = 6), and their care partners (n = 5), recruited from large Mid-Atlantic academic health centers. Most older adults were 76–85, White, male, with ≥5 years of hearing loss, and cared for by a spouse. Providers were predominantly internal medicine physicians with ≥5 years’ experience. Interviews explored four domains: 1) hearing loss impact on life, 2) caregiving perspectives, 3) hearing care needs, and 4) system-level implementation. All participants acknowledged hearing’s value for health and function but recognized cognitive and other concerns often took precedence during visits. Still, older adults and care partners preferred providers inquire about hearing to support connection and daily function. All desired improved interprofessional communication to ease self-advocacy and education burdens. Providers described hearing health as a “black box,” limiting conversation engagement. They expressed interest in accessible educational materials and structured resources to support patient conversations but noted limited time to address hearing during visits. Findings highlight opportunities for practical strategies and interventions to relieve provider burden and improve hearing care integration in primary care.

